# Supercapacitive microbial fuel cell: Characterization and analysis for improved charge storage/delivery performance

**DOI:** 10.1016/j.biortech.2016.06.105

**Published:** 2016-10

**Authors:** Jeremiah Houghton, Carlo Santoro, Francesca Soavi, Alexey Serov, Ioannis Ieropoulos, Catia Arbizzani, Plamen Atanassov

**Affiliations:** a Department of Chemical & Biological Engineering, Center for Micro-Engineered Materials (CMEM), University of New Mexico, Albuquerque, NM 87131, USA; b Department of Chemistry “Giacomo Ciamician”, Alma Mater Studiorum – Università di Bologna, Via Selmi, 2, 40126 Bologna, Italy; c Bristol BioEnergy Centre, Bristol Robotics Laboratory, Block T, UWE, Coldharbour Lane, Bristol BS16 1QY, UK; d Biological, Biomedical and Analytical Sciences, UWE, Coldharbour Lane, Bristol BS16 1QY, UK

**Keywords:** Microbial fuel cell (MFC), Supercapacitor (SC), Electrode area, Linear model, Power performance

## Abstract

Supercapacitive microbial fuel cells with various anode and cathode dimensions were investigated in order to determine the effect on cell capacitance and delivered power quality. The cathode size was shown to be the limiting component of the system in contrast to anode size. By doubling the cathode area, the peak power output was improved by roughly 120% for a 10 ms pulse discharge and internal resistance of the cell was decreased by ∼47%. A model was constructed in order to predict the performance of a hypothetical cylindrical MFC design with larger relative cathode size. It was found that a small device based on conventional materials with a volume of approximately 21 cm^3^ would be capable of delivering a peak power output of approximately 25 mW at 70 mA, corresponding to ∼1300 W m^−3^.

## Highlights

• Supercapacitive MFCs with various anode and cathode dimensions are investigated.
• Cathode is limiting bottle supercapacitive MFC performance.
• Increase in cathode area led to decrease in ohmic resistances and increase in capacitance.
• The performance of a hypothetical cylindrical MFC is linearly modelled.
• A 21 cm^3^ cylindrical MFC can deliver a peak power of 25  mW at 70  mA and 1300 W m^−3^.

## 1 Introduction

Microbial fuel cell (MFC) technology has been an area of interest over the past few decades as a potential source for sustainable alternative energy generation and simultaneous wastewater treatment (Pandey et al., 2016). Although power production of MFCs has increased greatly since the late 90’s, MFCs still deliver current/power densities that are approximately three orders of magnitude lower than those of methanol or hydrogen based fuel cells (Logan, 2009). These low current densities make it difficult to employ MFCs to directly power devices which require high energy output. Improving the power quality delivered by MFCs is a key challenge in the development of this technology.

Microbial fuel cells utilize innate bacterial respiratory processes to convert organic materials to usable energy through the process of extracellular electron transfer. Electro-active bacteria oxidize organic substrates and electrons are conducted through the bacterial membrane to an extracellular electron acceptor using specialized proteins (c-type cytochromes) and appendages (nanowires) that are present on the bacterial surface (Busalmen et al., 2008, Gorby et al., 2006, Logan, 2009). In MFCs, a conductive anode serves as the final electron acceptor in the bacterial respiratory process. Conductive 3-D carbonaceous (Chen et al., 2012, Wei et al., 2011, Liu et al., 2012) or metallic (Dumas et al., 2007, Guerrini et al., 2014, Baudler et al., 2015) materials have been used as anode electrodes. The electrons flow through a circuit that is terminated with the reduction of oxygen at the cathode, creating a gradient in electrical potential and generating current. Oxygen is commonly used as the oxidant at the cathode due to its high electrochemical potential and its environmental availability. At near neutral pH, the cathodic reaction has a large overpotential, so a catalyst is necessary to complete the reaction (Zhao et al., 2006, Erable et al., 2012). Typically, this is accomplished through the use of platinum or platinum group metals (PGMs), enzymes, bacteria, high surface area carbon materials, or high surface area carbon materials with PGM-free catalyst.

While platinum is one of the most effective materials currently known for the electrocatalytic oxygen reduction, it is very cost prohibitive resulting in 47% of the capital cost of the device (Rozendal et al., 2008). Furthermore, platinum catalysts are also subject to poisoning in the conditions present in MFC environments, leading to reduced efficiency over time (Santoro et al., 2016a). In this work, we utilize a platinum group metal-free organic catalyst (Fe-AAPyr) to catalyze the oxygen reduction reaction (ORR). Fe-AAPyr is competitive with platinum-based catalysts with the advantage of being much more cost effective, sustainable and less prone to catalyst poisoning (Santoro et al., 2016a).

The maximum theoretical cell voltage, V_max,theoretical_, of an MFC can be calculated by considering the equilibrium potentials of the anode and cathode reactions (E_cathode_ and E_anode_ in V vs. SHE):

(1) $${\text{V}}_{\text{max,theoretical}}={\text{E}}_{\text{cathode}}-{\text{E}}_{\text{anode}}=0.805\;\text{V}-0.300\;\text{V}=1.105\;\text{V}$$

The above equation assumes that acetate is used as a fuel source (16.9 mM) for the anode at pH = 7 and oxygen, at a partial pressure of 0.2 atm, is the oxidant for the cathode (Fradler et al., 2014). During operation, losses occur as a result of ohmic, activation, and mass-transport limitations, resulting in lower cell potentials of around 0.3–0.5 V. These voltage levels are insufficient to operate low-power consuming devices such as microprocessors (270 uA, 2.2 V), LEDs (10–20 mA, 2 V), or photodiodes (10 mA, 3.3 V) (Fradler et al., 2014). Various approaches have been explored for improving cell potential and power output in MFCs including: stacking of individual MFCs with series and parallel connections (Ieropoulos et al., 2008, Ledezma et al., 2013), maximum power point tracking (MPPT) techniques (Park and Ren, 2012), and the use of external capacitors with DC/DC converters (Dewan et al., 2009, Rozendal et al., 2008, Wang et al., 2015).

Electrochemical supercapacitors (SCs) are an attractive energy storage technology that is capable of storing and delivering energy at high current and power densities with little variation in performance over the course of millions of charge/discharge cycles (Conway, 1999). In addition, SCs offer the advantage of duty cycles more compatible with BES technologies, whereby the charge/discharge cycles can be within minutes, rather than hours, days or months, which is the case for conventional batteries. SCs differ from conventional capacitors in that they do not make use of a solid dielectric material. Instead, they rely on the principles of electric double-layer capacitance and/or pseudocapacitance as the charge storage mechanisms (Conway, 1999).

As stated above, external SCs have been utilized as an energy storage system to harvest the low power produced by MFCs and to deliver higher current pulses in order to power small electronic devices (Dewan et al., 2009, Ieropoulos et al., 2010, Ieropoulos et al., 2013, Wang et al., 2015). Furthermore, it has been demonstrated that more energy can be harvested by operating MFCs intermittently rather than continuously. Dewan et al. showed a 111% increase in power by intermittent operation of the MFC connected to a SC when compared to continuous operation (Dewan et al., 2009, Ieropoulos et al., 2016, Papaharalabos et al., 2013). Another approach to improve power quality is the utilization of the inherent capacitive features of MFC electrodes. MFCs and SCs both utilize high surface area carbon as their electrode material. Recently, efforts have been made to integrate capacitive materials with MFC electrodes in order to improve power quality and charge storage capabilities (Deeke et al., 2015). In 2005, Ieropoulos et al. first demonstrated that biofilms in MFCs were capable of storing electrons when the device was left in open circuit for an extended period of time, providing higher power upon reconnection of the circuit (Ieropoulos et al., 2005). It has been shown that cytochromes present within MFC biofilms exhibit pseudocapacitive behavior and can act as electron sinks (Esteve-Núñez et al., 2008, Schrott et al., 2011, Uría et al., 2011).

Formation of a Helmholtz layer by electrolyte ion adsorption at the MFC/electrode interfaces further contributes to the observed capacitance of the cell (Fradler et al., 2014). Fradler et al. showed that double layer capacitance contributed approximately ten times the capacitance of the biofilm in a tubular MFC which was shown to achieve charge storage capacities comparable to SCs with minimal current leakage (Fradler et al., 2014). An integrated self-charging supercapacitive MFC has been constructed by integrating an additional high surface area carbon brush short-circuited with the cathode and operating an MFC in a controlled manner (Santoro et al., 2016b). The additional electrode (AdE) confers increased surface area available for formation of a Helmholtz double layer, thus increasing the device’s capacitance. The AdE also leads to lower observed ohmic resistance during galvanostatic (GLV) discharge of the microbial supercapacitor. This design significantly improves recharge times of the system when compared to designs that incorporate external capacitors, allowing for more frequent use of the accumulated energy (Santoro et al., 2016b).

It was previously shown that the increase in cathode area affected positively on the performance output of the MFCs (Cheng and Logan, 2011, Kim et al., 2015). In the present study, we investigate a supercapacitive MFC (SC-MFC) system and the effect of relative anode and cathode size on the overall performance of the system. We use the experimental data from these experiments to construct a simple predictive linear model for a hypothetical SC-MFC with a cylindrical design in order to forecast performance of a larger scale device. We demonstrate that the performance of a SC-MFC based on conventional materials can be improved to levels suitable for powering practical electronic devices by optimizing design parameters.

## 2 Materials and method

### 2.1 MFC configuration

A single chamber glass bottle microbial fuel cell design with a volume of 125 mL was used to investigate the effect of relative anode and cathode geometric area on supercapacitive MFC (SC-MFC) performance (Fig. S1). The cell consisted of a Pyrex glass bottle modified with two lateral glass tubes to serve as attachment sites for cathode electrodes. The MFC was operated in a membraneless configuration with the anode fully immersed in the solution with air-breathing cathodes. One face of the cathode was exposed to the electrolyte and the other was exposed to the air. The effect of changing the relative area of the anode and cathode of the MSC was investigated.

### 2.2 Anode construction

Carbon brush electrodes (Millirose, USA) were employed as the anode material for all experiments. The carbon brushes used had a diameter of 3 cm and a length of 3 cm, giving a projected surface area of 9 cm^2^. Prior to our experiments, all anodes were pre-colonized with electro-active bacterial biofilms by incubation in a mixture (by volume) of 50% activated sludge (obtained from Albuquerque Southeast Water Reclamation facility, Albuquerque, NM) and 50% buffer solution composed of 0.1 M KCl and 0.1 M potassium phosphate buffer (pH = 7.5). The same anodes have been used for previous experiments (Santoro et al., 2016b, Soavi et al., 2016). Additional carbon brushes were added to the cell to investigate the effects of increased anode surface area. All carbon brushes were colonized with electroactive bacteria as described above (Santoro et al., 2016a, Santoro et al., 2016b).

### 2.3 Cathode construction

Air-breathing gas diffusion electrodes were used for the cathodes. The electrode consisted of a hydrophobic-hydrophilic gradient of carbon infused with iron-aminoantipyrine (Fe-AAPyr), a PGM-free catalyst (Serov et al., 2014). Cathodes were constructed by pressing carbon-based materials onto stainless steel mesh used as current collector. First, carbon black teflonized with 50 wt% of PTFE (XC50) with a loading of 30 ± 1 mg cm^−2^ was mixed using a blade-type coffee grinder and pressed in a circular pellet die at 2 metric tons (mT) for 5 min. A secondary layer was added consisting of 20 ± 1 mg cm^−2^ carbon black teflonized with 35 wt% of PTFE (XC35) mixed with 2 ± 0.1 mg cm^−2^ Fe-AAPyr and pressed in the pellet die at 2 mT for 5 min at room temperature. The preparation of Fe-AAPyr was based on the sacrificial support method (SSM) that has been previously described (Serov et al., 2014). Cathodes were attached to the MFC using stainless steel screw clamps and rubber gaskets.

The cathode area was modified using rubber gaskets with circular holes with diameters of 1.2 cm and 1.8 cm. The area of the circular holes was 1.13 cm^2^ (d = 1.2 cm) and 2.54 cm^2^ (d = 1.8 cm) respectively. The different cathode areas used in the experiments were: i) 2.54 cm^2^ (single cathode), ii) 3.67 cm^2^ (two cathodes with one 2.54 cm^2^ gasket and one 1.13 cm^2^ gasket), iii) 5.09 cm^2^ (two cathodes each with a 2.54 cm^2^ gasket).

### 2.4 Electrochemical measurements

**Fig. 1:**
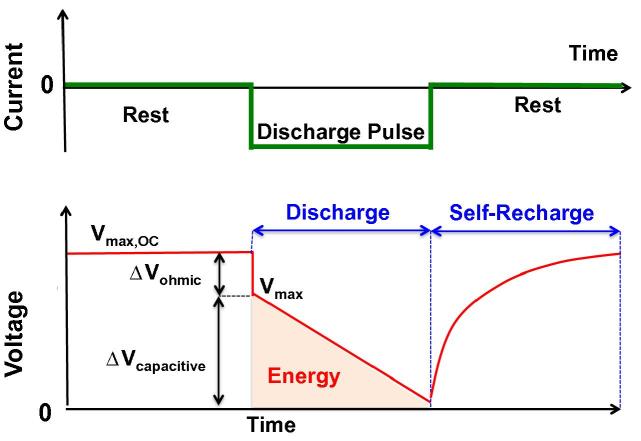
Schematic representation of the rest-galvanostatic discharge – rest sequence. No current is applied during the rest period.

Electrochemical measurements were carried out using a BioLogic SP-50 potentiostat using a three-electrode setup with an Ag/AgCl (3 M KCl, +210 mV vs. SHE) reference electrode. The cell was left in open circuit until a steady state potential was attained. Then, a sequence following the order: *rest – galvanostatic discharge – rest*, was repeated. The galvanostatic discharge was run at different current levels (i_pulse_) (Fig. 1) with pulse times of 2 s and 10 ms while monitoring the anode and cathode potentials by the use of a reference electrode (Ag/AgCl 3 M KCl). Following each pulse, the SC-MFC was allowed to rest (no current applied, the circuit is opened) until the potential returned to the original open circuit voltage, (V_max,OC_). During this time, the electrode potentials are restored to their equilibrium values exhibited before the pulse, recharging the SC-MFC independently of an external power source (Santoro et al., 2016b).

During the GLV discharge, an initial drop in cell voltage (from V_max,OC_ to a lower value, V_max_) is observed This initial drop in potential (V_max,OC_–V_max_ = ΔV_ohmic_) is directly related to the equivalent series resistance (ESR) of the cell. The ΔV_ohmic_ includes contributions from the electrolyte as well as the electrodes. The relationship between ESR and ΔV_ohmic_ is demonstrated by Eq. (2):

(2) $$\mathrm{ESR}=\frac{\Delta V_{\mathrm{ohmic}}}{i_{\mathrm{pulse}}}$$

The ESR of the cell can be further analyzed to investigate the individual contributions of the anode and cathode by examining each electrode profile under the GLV discharges. The ohmic losses observed at each electrode can be used to estimate the anode (R_A_) and cathode resistances (R_C_)_._ Specifically, R_A_ and R_C_ are obtained by dividing the electrode ohmic losses per i_pulse_. The reference electrode is centered between the anode and the cathode and the bulk electrolyte resistance is assumed to be negligible. The cell ESR is related to R_A_ and R_C_ by Eq. (3):

(3) $$\mathrm{ESR}=R_{A}+R_{C}$$

The capacitance of the SC-MFC influences the rate at which the cell voltage (ΔV_capacitive_) decreases during the GLV discharge, following the initial ohmic drop. The slope of the GLV discharge curve over time (dV/dt) is inversely related to the capacitance of the cell. Capacitance (C) was calculated using Eq. (4):

(4) $$C_{\mathrm{cell}}=\frac{i_{\mathrm{pulse}}}{\frac{\mathrm{dV}}{\mathrm{dt}}}$$

Anode capacitance (C_A_) and cathode capacitance (C_C_) were calculated by analyzing the slopes of the corresponding electrode potentials over time. The total cell capacitance (C_cell_), is related to C_A_ and C_C_ by Eq. (5):

(5) $${\text{C}}_{\text{cell}}={\left(\frac{1}{{\text{C}}_{\text{A}}}+\frac{1}{{\text{C}}_{\text{C}}}\right)}^{-1}$$

Maximum power output (P_max_) was calculated for each SC-MFC configuration by multiplying the maximum cell voltage after the pulse (V_max_) by the pulse current:

(6) $$P_{\max}=i_{\mathrm{pulse}}\times V_{\max}$$

Since this calculation does not account for the capacitive decrease of cell voltage (ΔV_capacitive_) observed during discharge of the SC-MFC, the P_max_ value is higher than the actual power delivered by the device over the pulse duration. This pulse power (P_pulse_) is calculated on the basis of the energy delivered during the pulse (E_pulse_), which in turn is calculated by Eq. (7):

(7) $$E_{\mathrm{pulse}}=i_{\mathrm{pulse}}\int_{0}^{t}\mathrm{Vdt}$$

where t is the discharge time. The pulse power is obtained by Eq. (8):

(8) $$P_{\mathrm{pulse}}=\frac{E_{\mathrm{pulse}}}{t}$$

## 3 Results and discussion

### 3.1 Effect of cathode geometric area on SC-MFC performance

**Fig. 2:**
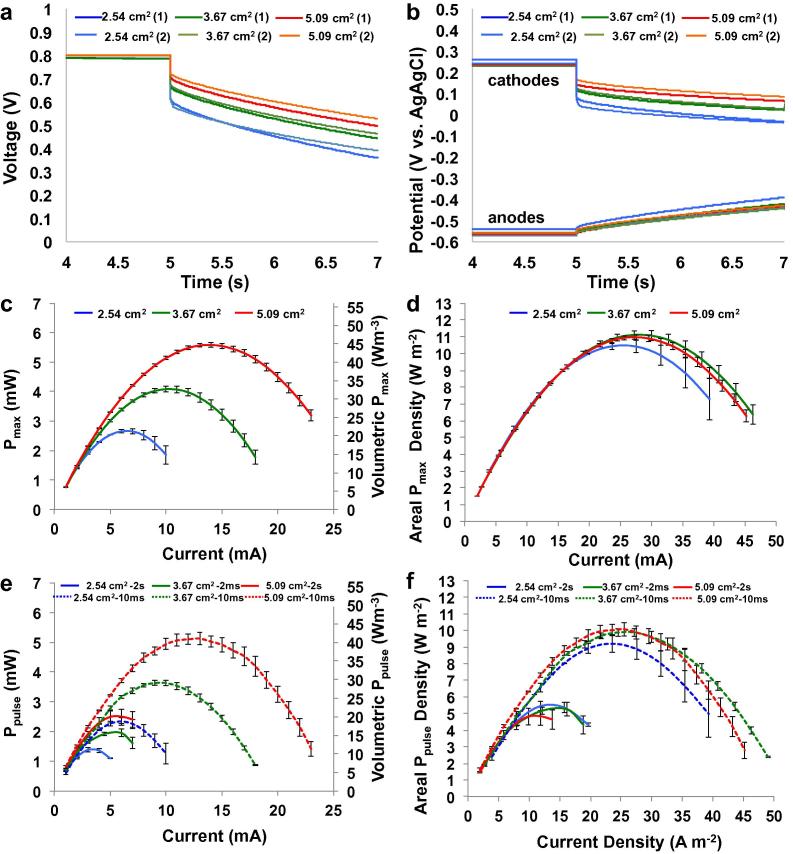
Cell voltage (a) and electrode potential (b) profiles under 2 s pulses at 3 mA of SC-MFCs after 5 s rest. P_max_ (c and d) and P_pulse_ (e and f) vs. I plots for SC-MFC with different cathodes. Cathode area is 2.54 cm^−2^ (blue), 3.67 cm^−2^ (green) and 5.09 cm^−2^ (red). Volumetric power densities (c and e) are normalized to the cell volume (125 mL). Areal power and current densities (d and f) are normalized to the cathode geometric area. (For interpretation of the references to colour in this figure legend, the reader is referred to the web version of this article.)

Fig. 2 reports the results of the GLV discharge of SC-MFCs featuring cathodes of various areas. The results are summarized in Table 1. Cell voltage and electrode potential profiles for 2 s discharges at 3 mA are shown in Fig. 2a and b. Cell voltage and electrode potential profiles for 10 ms discharges at 3 mA are shown in Fig. S2. As cathode area doubled from 2.54 cm^2^ to 5.09 cm^2^, ΔV_ohmic_ decreased by approximately 47%. The values for the overall ΔV_ohmic_ were measured to be 176 ± 1.5 mV for the 2.54 cm^2^ cathode, 115 ± 3.5 mV for the 3.67 cm^2^ cathode, and 91 ± 3 mV for the 5.09 cm^2^ cathode area. The ESR for each cell was calculated to be 58.6 ± 0.3 Ω, 38.1 ± 0.9 Ω, and 30.5 ± 0.9 Ω respectively. Cell capacitance (C_cell_) also increased with increasing cathode geometric area, with measured values of 24 ± 2 mF, 27 ± 0.1 mF and 30 ± 1.4 mF respectively (Fig. 2a and Table 1).

Fig. 2b shows that the cathode is the main contributor to ΔV_ohmic_ and ESR. Cathode resistances (R_C_) of 57.1 ± 2.6 Ω (2.54 cm^2^), 35.7 ± 1.4 Ω (3.67 cm^2^), and 27.9 ± 4.2 Ω (5.09 cm^2^) were observed. These values correspond to a cathode resistance normalized to electrode geometric area (R_C_′) of approximately 140 Ω cm^2^. Cathode capacitance (C_C_) increased with increasing cathode area, with recorded values of 51.3 ± 1.9, 61.5 ± 0.5, and 73.2 ± 1.3 mF. This translates to a cathode capacitance normalized to electrode geometric area (areal capacitance density, C_C_′) of ∼17 mF cm^−2^ (Table 1). Anode resistance and capacitance remained constant at approximately 0.5 Ω and 48 mF (Table 1).

Fig. 2c shows the P_max_ values of the three cells as calculated by Eq. (4) for various discharge currents. P_max_ increased significantly with increasing cathode geometric area. Doubling the cathode’s geometric area resulted in a 113% increase in P_max_ indicating quasi-linear positive dependence between cathode area and P_max_. Recorded P_max_ values were 2.65 ± 0.05 mW (i = 6 mA) for the SC-MFC with a 2.54 cm^2^ cathode area, 4.08 ± 0.1 mW (i = 10 mA) for the SC-MFC with a 3.67 cm^2^ cathode area, and 5.58 ± 0.08 mW (i = 14 mA) for the SC-MFC with a cathode area of 5.09 cm^2^ (Fig. 2d). These values correspond to volumetric power densities of 21.2 ± 0.4 W m^−3^, 32.64 ± 0.8 W m^−3^, and 44.64 ± 0.64 W m^−3^ respectively (based on 125 mL volume) (Fig. 2c).

Fig. 2d reports the P_max_ vs i_pulse_ curves with power and current normalized to the cathode geometric area, i.e. in terms of areal power and current densities. It was found that the areal P_max_ density was similar among the different SC-MFCs. Values ranged from 10.4 ± 0.2 W m^−2^ (at 23.6 A m^−2^) for the 2.54 cm^2^ cathode area to 11.0 ± 0.16 W m^−2^ (at 27.5 A m^−2^) for the 5.09 cm^2^ cathode area. Similar measured power density values indicated a roughly linear positive relationship between the power generated and the cathode area. Therefore, an increase in cathode area led to a roughly linear increase in power performance.

Fig. 2e and f report the pulse power (P_pulse_) delivered over 10 ms and 2 s pulses. As expected, the longer pulse time led to smaller power produced due to the capacitive decrease of cell voltage (ΔV_capactive_). In agreement with P_max_ data (Fig. 2c), the increase in cathode area led to higher power (Fig. 2e). For pulse durations of 10 ms, the SC-MFC with smaller cathode area delivered 2.3 ± 0.13 mW at 6 mA (19 ± 1 W m^−3^). Doubling the cathode’s geometric area resulted in ∼118% higher power (5.1 ± 0.21 mW, 41 ± 1.7 W m^−3^) at 13 mA for 10 ms discharge pulse durations. A similar trend was observed for 2 s pulse durations. The SC-MFC with cathode area of 2.54 cm^−2^ generated 1.38 ± 0.07 mW at 3 mA (11 ± 0.56 W m^−3^), the SC-MFC with 5.09 cm^−2^ cathode area increased the power output (2.5 ± 0.25 mW) under the same conditions. Volumetric power increased by approximately 80% (20 ± 2 W m^−3^) by doubling the cathode area (Fig. 2e).

## 4 Effect of anode geometric area on SC-MFC performance

**Fig. 3:**
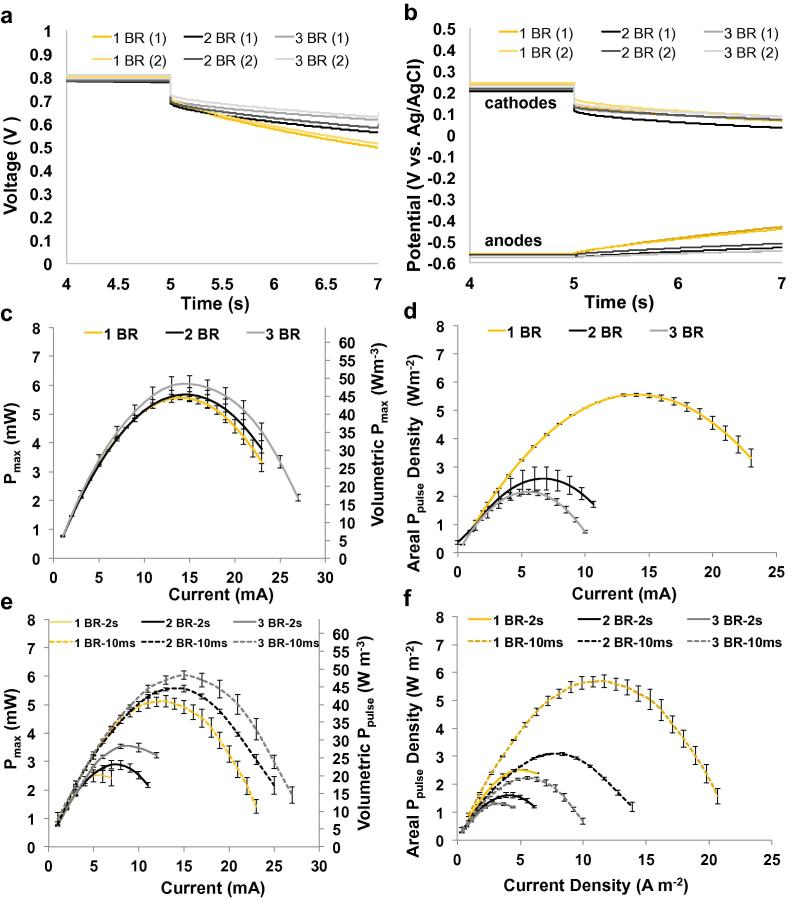
Cell voltage (a) and electrode potential (b) profiles under 2 s pulses at 3 mA after 5 s rest. P_max_ (c and d) and P_pulse_ (e and f) vs. I for SC-MFC with different number of brush anodes. The projected anode areas are 9 cm^−2^ (yellow), 18 cm^−2^ (black) and 27 cm^−2^ (grey). Volumetric power densities (c and e) are normalized to the cell volume (125 mL). Areal power and current densities (d and f) are normalized to the projected anode area (9 cm^2^ brush^−1^). (For interpretation of the references to colour in this figure legend, the reader is referred to the web version of this article.)

Fig. 3a and b show discharge profiles for cell voltage and individual electrode potentials at discharge times of 2 s at a pulse current of 3 mA for SC-MFCs with different anode geometric areas (number of anode brushes). Cell voltage and cell potentials at discharge time of 10 ms (i_pulse_ 3 mA) are reported on the Fig. S3. The results of the GLV curve analyses are reported in Table 1. In these experiments, the cathode area was maintained at 5.09 cm^2^, as it showed the highest performance from previous experiments. As mentioned in the description of materials, each anode was considered to have a projected area of 9 cm^2^ per brush.

The ESR of the cell was measured as 30.5 ± 0.9 Ω (one brush), 29.4 ± 0.5 Ω (two brushes), and 26.8 ± 0.5 Ω (three brushes) respectively (Fig. 3a and Table 1). The anode electrode contribution to ohmic losses (R_A_ of 0.64 ± 0.2 Ω) is very low compared to the ohmic losses contributed by the cathode electrode (Fig. 3b). Anode resistance remained relatively constant under all three experimental conditions, suggesting that the bulk electrolyte resistance, which remained constant in all three cells, is the main contributor to anode resistance.

Total cell capacitance (C_cell_) increased when the number of brushes was increased (Fig. 3a), with measured values of 30 ± 1.4, 50 ± 3.7 and 63 ± 1.9 mF (Table 1). This trend is directly related to C_A_, which increased from 50 ± 4 mF to 121 ± 14 mF and 194 ± 18 mF (Fig. 3b), in agreement with a capacitance of ∼53 mF per brush (Table 1).

The P_max_ vs. I plots are shown in Fig. 3c. P_max_ was relatively consistent between all three cells (Fig. 3c) with a measured value of ∼5.6–6.0 mW (45–48 Wm^−3^). A slightly higher value was obtained for the cell with three brushes and is likely due to the lower observed ESR of the cell. When P_max_ was represented as areal power density (Fig. 3d), the SC-MFC with three brushes showed the lowest value (2.2 ± 0.1 W m^−2^) followed by the SC-MFC with two anode brushes (3.16 ± 0.05 W m^−2^) and the highest areal power density was observed with just one anode brush (6.2 ± 0.19 W m^−2^). This trend shows that increasing anode area does not improve the areal power performance of the system, and further demonstrates that the cathode is the limiting component of the system.

The pulse power for 10 ms and 2 s pulse durations is reported in (Fig. 3e and f). Unlike the trend observed in the P_max_ plots (Fig. 3c), the P_pulse_ curves expressed in mW and as volumetric power (W m^−3^) show a clear increasing trend with the anode size (Fig. 3e). During the 10 ms discharge, the peak power achieved for SC-MFC with three anode brushes was 6.03 ± 0.16 mW (48 ± 1.3 W m^−3^) followed by SC-MFC with two anode brushes (5.6 ± 0.11 mW, 44.5 ± 0.88 W m^−3^) and the one with just single brush (5.12 ± 0.22 mW, 41 ± 1.68 W m^−3^) (Fig. 3e). The 2 s discharge pulses show a more pronounced difference between the three cells. The difference can be attributed to the increase in C_cell_. The maximum P_pulse_ values (t_pulse_ of 2 s) for each SC-MFC configuration were as follows: 3.53 ± 0.09 mW (28.2 ± 0.72 W m^−3^) for the cell with three anode brushes, 2.9 ± 0.15 mW (23 ± 1.2 W m^−3^) for the cell with two anode brushes, and 2.51 ± 0.25 mW (20 ± 3 W m^−3^) for the cell with a single anode brush (Fig. 3e). Areal power density (W m^−2^) decreased with increasing anode area, indicating that increasing anode geometric area did not improve performance, and that cathode is the limiting factor of the system in terms of performance (Fig. 3f).

### 4.1 Simple linear predictive model

**Fig. 4:**
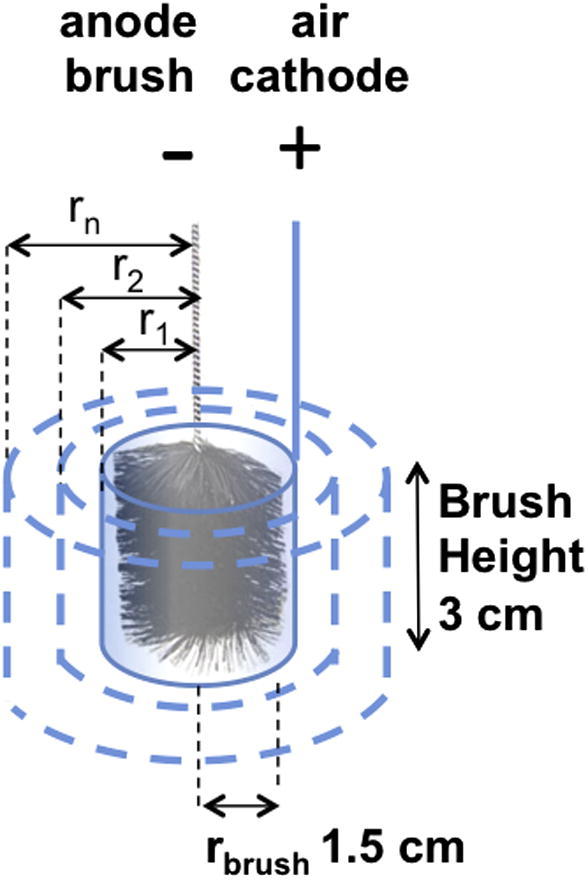
Schematic of the cylindrical SC-MFC used for predictive model.

The experimental data described above suggest that the SC-MFC performance can be significantly improved by properly balancing the anode and cathode geometric areas. Cathode is the limiting component of system performance as demonstrated by the linear relationship between cathode area and power generated. We have shown that increasing cathode geometric area is a viable strategy for improving SC-MFC power output by increasing the capacitance and decreasing the ESR. The most efficient design that minimizes SC-MFC volume and maximizes cathode area is a cylinder with the air cathode comprising the cylinder wall surrounding the anode brush and adequately spaced in order to avoid short circuit (Fig. 4).

We devised a simple linear model in order to predict the performance of the cylindrical SC-MFCs with various radial diameters using experimental GLV data obtained at 3 mA and 2 s (Table 1). The minimum cell volume is limited by the size of the anode brush, which has a radius (r_brush_) of 1.5 cm and a height (h) of 3 cm, resulting in a volume of 21.3 cm^3^ (V = π r_brush_^2^ h). The cathode area for a cylinder of these dimensions is 28.4 cm^2^ (cathode area = 2 π r h) (Table 2). Since the height of the brush (h) is constant, the cathode area scales with the cylinder radius by 2 π r, reaching a maximum value of 94.2 cm^2^ at r = 5 cm. We calculated the C_cell_ and ESR of hypothetical cylindrical cells with radii (r) ranging between 1.5 and 5 cm as described in Table 2 and by the following Eqs. (9), (10), (11), (12). Since all cells in the model utilize a single anode brush, anode capacitance (C_A_) remained at a value of 53 mF (Table 1). Cathode capacitance (C_C_) was calculated by utilizing a cathode areal capacitance density (C_C_′) of 17 mF cm^−2^ using Eq. (9):

(9) $$C_{c}=C_{C}^{\prime }\times 2\pi \mathrm{rh}$$

C_C_ values ranged from 480 mF for r = 1.5 cm to 1600 mF for r = 5 cm. The calculated C_C_ values are more than one order of magnitude higher than C_A_, therefore total cell capacitance (C_cell_) is roughly unaffected by cathode geometric area. Indeed, substituting Eq. (9) into Eq. (5), C_cell_ is represented by Eq. (10):

(10) $$C_{\mathrm{cell}}={\left(\frac{1}{C_{A}}+\frac{1}{C_{C}^{\prime }\times 2\pi \mathrm{rh}}\right)}^{-1}={\left(\frac{1}{53}+\frac{1}{320\;r}\right)}^{-1}$$

C_cell_ is ∼50 mF for all cells and it can be improved by increasing the height of the anode brush.

The ESR of the cell is directly related to the cylinder radius and cathode area. ESR was calculated as the sum of the anode resistance (R_A_) and cathode resistance (R_C_) according to Eq. (3). The contribution of the electrolyte to ESR was considered to be negligible. R_A_ was set at a constant value of 0.5 Ω (Table 1). R_C_ was obtained for each cylindrical radius using the R_C_′ value of 140 Ω cm^2^ (Table 1) using Eq. (11):

(11) $$R_{C}=\frac{R_{C}^{\prime }}{2\pi \mathrm{rh}}$$

Substituting Eq. (10) into Eq. (3), ESR is represented by Eq. (12):

(12) $$\mathrm{ESR}=R_{A}+\frac{R_{C}^{\prime }}{2\pi \mathrm{rh}}$$

Table 2 shows that R_C_ decreases from a value of 5 Ω to 1.5 Ω when r increases from 1.5 cm to 5 cm, resulting in ESR values of 5.5 Ω to 2 Ω respectively.

Calculated C_cell_ and ESR values were used to simulate GLV discharge curves for cylindrical SCMFCs with different radii at various currents (i) using Eqs. (13), (14):

(13) $$V_{\mathrm{cell}}=V_{\max}-\Delta V_{\mathrm{ohmic}}-\Delta V_{\mathrm{capacitive}}$$

where $V_{\max}=0.8\;V\text{,}\;\Delta V_{\mathrm{ohmic}}=i\times \mathrm{ESR}\;\;\text{and}\;\Delta V_{\mathrm{capacitive}}=\frac{i\times t}{C_{\mathrm{cell}}}\text{,}$ therefore,

(14) $$V_{\mathrm{cell}}=0.8-i\times \mathrm{ESR}-\frac{i\times t}{C_{\mathrm{cell}}}$$

**Fig. 5:**
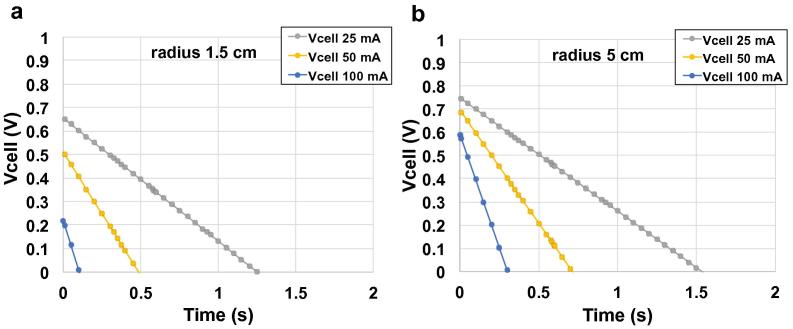
Cell voltage profiles of two cylindrical SC-MFCs with r = 1.5 cm (a) and 5 cm (b) calculated at i = 25, 50 and 100 mA by Eqs. (14), (15) on the basis of the values of ESR and C_cell_ reported in Table 2.

Fig. 5 shows the simulated discharge curves for two cells with r = 1.5 cm and 5 cm with discharge current i = 25, 50 and 100 mA. It can be seen that r has little effect on capacitance (slope), but significantly affects ΔV_ohmic_, which is related to the cell’s ESR (see Table 1).

The V_cell_ vs. time profiles were analyzed to calculate the maximum power, P_max_ by Eq. (15), and the energy E and power P delivered during a full discharge at different i by the Eqs. (6), (7):

(15) $$P_{\max}=i\times (V_{\max}-\Delta V_{\mathrm{ohmic}})=i\times (V_{\max}-i\times \mathrm{ESR})$$

**Fig. 6:**
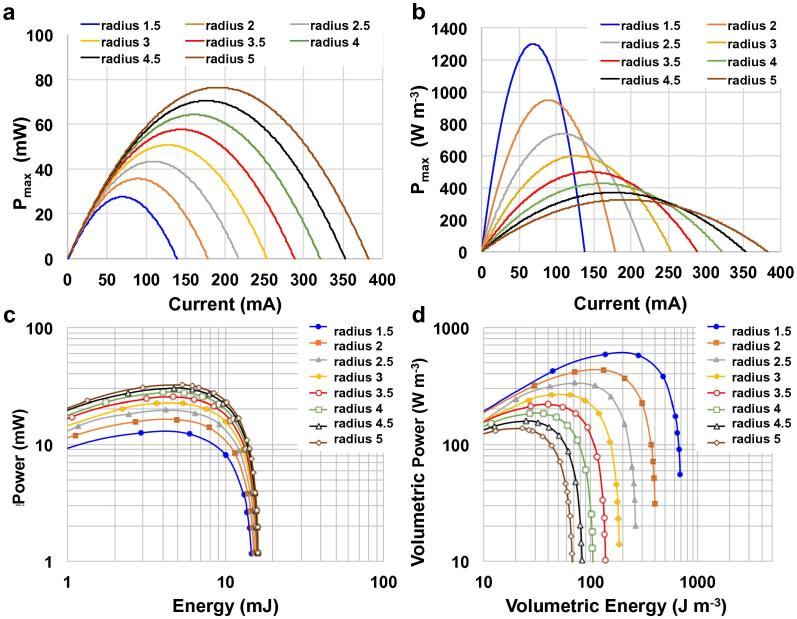
Projected P_max_ vs current plots (a and b) and Ragone plots (c and d) of cylindrical SC-MFCs with different radius (r).

Fig. 6 shows the P_max_ vs I plots calculated for cells of various radii. Fig. 6a shows that an increase in the diameter of the cylindrical SC-MFC improves power performance. The cell with a radius of 5 cm is capable of producing up to 80 mW at 200 mA discharges current. However, the increased cylinder diameter leads to a larger cell volume, and therefore has a negative impact on the volumetric P_max_ values. This is evident in Fig. 6b, where the highest power density of 1300 W m^−3^ is obtained at 70 mA with the smallest cell. It is worth noting, that despite a lower maximum power density of 320 W m^−3^, the biggest cell permits operation at higher discharge currents.

These projected performance levels have been calculated on the basis of 2 s discharges at 3 mA. The model could be further implemented by the use of parameterization data that refer to specific operative conditions of the SC-MFC, and which take into account the effects of discharge current and time on the capacitive response of the cell, which in turn is expected to increase at lower currents (from mA to tens of microA) and for longer periods (from seconds to minutes) (Conway, 1999).

In previous cases, single MFCs of 6.25 mL volume, produced 0.1 mW at 0.45 mA and 220 mV, but when two such units were connected together, a digital wristwatch was powered via an ultra-low power boost converter (Papaharalabos et al., 2013). In the second instance, individual 100 mL MFCs were generating between 1 and 2 mW continuously, and a 36-MFC module produced 40–60 mW continuously, which was part of a stack powering LED modules, (via a voltage regulator and an external supercapacitor), consuming approximately 1.5 W (Ieropoulos et al., 2016). The amount of absolute power generated by the larger cell (r = 5 cm, 80 mW) in this study is calculated to be at a cell voltage of 0.4 V. Although this is transient (i.e. not continuous) since it is generated as a pulse, it is still higher compared with the output levels of MFC modules previously reported to power practical applications (Ieropoulos et al., 2016, Papaharalabos et al., 2013). Hence, the data generated by this simple linear predictive model, suggest that with intermittent operation, the SC-MFC could easily power practical applications such as LEDs or other low-power dc applications.

The Ragone plots in Fig. 6c and d show the calculated values for energy and power for complete discharges, from 0.8 V to 0 V, at different currents. Fig. 6c shows the highest energy of ∼4 μWh (15 mJ) is delivered at the lowest currents for all the cells. This is due to the fact that at the lower currents, the cell voltage profile over time is mainly affected by the capacitance of the cell (C_cell_), which is nearly identical for all the cylinder diameters. At higher currents, the ohmic drop is larger, leading to lower quantities of delivered energy. This phenomenon is less prominent in the larger cells that exhibit lower ESRs. The highest power is 30 mW and is delivered by the SC-MFC with r = 5 cm under a 150 ms pulse at 150 mA. Fig. 6d confirms that the smallest cell exhibits the highest volumetric energy and power densities of 700 J m^−3^ (195 mW h m^−3^) and 600 W m^−3^.

## 5 Conclusions

Cathode geometric area is a critical design component towards the improvement of SC-MFCs power performance. By increasing cathode area, the internal resistance decreased substantially and the peak power of the device scaled roughly linearly. A simple linear model was developed to predict the performance of a cylindrical SC-MFC. The model demonstrates that a SC-MC design with a greater relative cathode area should greatly improve the system performance. Due to the increased cathode surface area imparted by this design, volumetric power output is forecast to improve by more than two orders of magnitude, with an anticipated maximum value of ∼1300 W m^−3^.

## Figures and Tables

**Table 1:** ESR and capacitance of the SC-MFC and electrode resistances and capacitances evaluated from the GLV discharge curves at 3 mA reported in Figs. 3a and b and 4a and b. R_A_′, R_C_′, C_A_′ and C_C_′ are the anode and cathode resistances and capacitances normalized to the electrode geometric areas.

n. anode brush	Anode brush area	Cathode area	ΔV_ohmic_	ESR	Rc	Rc′	R_A_	R_A_′
cm^2^	cm^2^	mV	Ω	Ω	Ω cm^−2^	Ω	Ω brush^−1^
1	9	2.54	176 ± 1.5	58.6 ± 0.3	57 ± 2.6	145	0.8 ± 0.9	0.8
1	9	3.67	115 ± 3.5	38.1 ± 0.9	36 ± 1.4	131	0.8 ± 0.7	0.8
1	9	5.09	91 ± 3	30.5 ± 0.9	28 ± 4.2	142	0.35 ± 0.3	0.35
1	9	5.09	91 ± 3	30.5 ± 0.9	28 ± 4.2	142	0.35 ± 0.3	0.35
2	18	5.09	88 ± 1	29.4 ± 0.5	29 ± 0.7	148	0.4 ± 0.1	0.20
3	27	5.09	81 ± 1.5	26.8 ± 0.5	26.4 ± 0.6	134	0.6 ± 0.3	0.20

Average						140		0.5

n. anode brush	Anode brush area	Cathode area	ΔV_capac._	C_cell_	Cc	Cc′	C_A_	C_A_′
cm^2^	cm^2^	mV	mF	mF	mF cm^−2^	mF	mF cm^−2^

1	9	2.54	250 ± 20	24 ± 2	51 ± 1.9	20.	46 ± 8.3	46
1	9	3.67	226.5 ± 0.7	26.5 ± 0.1	61.5 ± 0.5	17	46 ± 2.8	49
1	9	5.09	203 ± 9.2	30 ± 1.4	73 ± 1.3	14	50 ± 4	50
1	9	5.09	203 ± 9.2	30 ± 1.4	73 ± 1.3	14	50 ± 4	50
2	18	5.09	122 ± 9.2	50 ± 3.7	86 ± 18	17	121 ± 13.7	60
3	27	5.09	95 ± 2.9	63 ± 1.9	95 ± 1	19	194 ± 17.7	65

Average						17		53

**Table 2:** The figures of merit of cylindrical SC-MFCs with increasing radius (r).

Size		Eq.	r			
1.5 cm	3 cm	4 cm	5 cm
Anode	r_brush_ = 1.5 cm h = 3 cm					
Cathode	h = 3 cm area = 2 π r h, with r > r_brush_ lowest area = 28.3 cm^2^		28.3 cm^2^	56.5 cm^2^	75.5 cm^2^	94.2 cm^2^
Cell	h = 3 cm volume = π r^2^ h, with r > r_brush_ lowest volume = 21.2 cm^3^		21.2 cm^3^	85 cm^3^	150 cm^3^	235 cm^3^

*Capacitance*						
Anode, C_A_	53 mF		53 mF	53 mF	53 mF	53 mF
Cathode, C_C_	C_C_ = C_C_′ × 2 π r h with C_C_′ = 17 mF cm^−2^	(9)	480 mF	960 mF	1280 mF	1600 mF
Cell, C_cell_	${\left(\frac{1}{{\text{C}}_{\text{A}}}+\frac{1}{{\text{C}}_{{\text{C}}^{\prime }}\times 2\pi \mathrm{rh}}\right)}^{-1}=$$={\left(\frac{1}{53}+\frac{1}{320\text{r}}\right)}^{-1}$	(10)	48 mF	50 mF	51 mF	51 mF

*ESR*						
Anode resistance, R_A_	0.5 Ω		0.5 Ω	0.5 Ω	0.5 Ω	0.5 Ω
Cathode resistance, R_C_	$R_{C}=\frac{R_{C}^{\prime }}{2\pi \mathrm{rh}}$, with R_C_′ = 140 Ω cm^2^	(11)	5	2.5	1.9	1.5
Cell, ESR	ESR = R_A_ + R_C_ = R_A_ + $\frac{R_{C}^{\prime }}{2\pi \mathrm{rh}}=$$=0.5+\frac{8}{r}$	(12)	5.5	3	2.4	2
