# Supplementary material for: Supercapacitive microbial fuel cell: Characterization and analysis for improved charge storage/delivery performance
Source: Bioresour Technol. 2016 Oct;218:552–60. doi: 10.1016/j.biortech.2016.06.105 (PMC5001197; doi:10.1016/j.biortech.2016.06.105)
Supplement: Supplementary data [file mmc1.docx]

**Supercapacitive Microbial Fuel Cell: Characterization and analysis for improved charge storage/delivery performance**

Jeremiah Houghton^1^, Carlo Santoro^1^, Francesca Soavi^2^, Alexey Serov^1^, Ioannis Ieropoulos^3,4^, Catia Arbizzani^1^, *Plamen Atanassov^2^

^1^ Department of Chemical & Biological Engineering, Center for Micro-Engineered Materials (CMEM), University of New Mexico, Albuquerque, NM 87131, USA.

^2^ Department of Chemistry “Giacomo Ciamician”, Alma Mater Studiorum - Università di Bologna, Via Selmi, 2, 40126 Bologna, Italy.

^3^ Bristol BioEnergy Centre, Bristol Robotics Laboratory, Block T, UWE, Coldharbour Lane, Bristol BS16 1QY, UK

^4^ Biological, Biomedical and Analytical Sciences, UWE, Coldharbour Lane, Bristol BS16 1QY, UK

***corresponding author**

Plamen Atanassov, Center for Micro-Engineered Materials (CMEM), Department of Chemical & Biological Engineering, University of New Mexico, Albuquerque, NM 87131, USA, e-mail: plamen@unm.edu

**Figure S1. SC-MFC device.**

**Figure S2. Cell voltage (a) and electrode potential (b) profiles under 10 ms pulses at 3 mA for SC-MFCs with different cathode area.**

**Figure S3. Cell voltage (a) and electrode potential (b) profiles under 10 ms pulses at 3 mA for SC-MFCs with different anode area.**

**Table S1. P_max_, P_pulse_ (2 s), P_pulse_ (10 ms) of different SC-MFCs with different anode and cathode areas.**

| **n. anode brush** | **Anode brush area** | **Cathode area** | **P_max_** | **P_max_** | **P_max_** |
| --- | --- | --- | --- | --- | --- |
|  |  |  |  |  |  |
|  | **cm^2^** | **cm^2^** | **mW** | **W m^-3^** | **W m^-2^** |
| 1 | 9 | 2.54 | 2.65±0.05 | 21.2±0.4 | 10.4±0.2 |
| 1 | 9 | 3.67 | 4.1±0.1 | 32.64±0.8 | 11.1±0.27 |
| 1 | 9 | 5.09 | 5.58±0.08 | 44.6±0.64 | 11.0±0.16 |
| 1 | 9 | 5.09 | 5.58±0.09 | 44.6±0.64 | 6.2±0.09 |
| 2 | 18 | 5.09 | 5.68±0.08 | 45.4±0.72 | 3.16±0.05 |
| 3 | 27 | 5.09 | 6.1±0.27 | 48±2.16 | 2.2±0.1 |
|  |  |  |  |  |  |
| **n. anode brush** | **Anode brush area** | **Cathode area** | **P_pulse_ (2 s)** | **P_pulse_ (2 s)** | **P_pulse_ (2 s)** |
|  |  |  |  |  |  |
|  | **cm^2^** | **cm^2^** | **mW** | **W m^-3^** | **W m^-2^** |
| 1 | 9 | 2.54 | 1.38±0.07 | 11±0.56 | 5.43±0.28 |
| 1 | 9 | 3.67 | 2.0±0.13 | 16±1.04 | 5.31±0.35 |
| 1 | 9 | 5.09 | 2.5±0.25 | 20±2 | 4.93±0.49 |
| 1 | 9 | 5.09 | 2.5±0.25 | 20±3 | 2.79±0.28 |
| 2 | 18 | 5.09 | 2.9±0.15 | 23±1.2 | 1.61±0.08 |
| 3 | 27 | 5.09 | 3.53±0.09 | 28.2±0.72 | 1.31±0.03 |
|  |  |  |  |  |  |
| **n. anode brush** | **Anode brush area** | **Cathode area** | **P_pulse_**  **(10 ms)** | **P_pulse_**  **(10 ms)** | **P_pulse_**  **(10 ms)** |
|  |  |  |  |  |  |
|  | **cm^2^** | **cm^2^** | **mW** | **W m^-3^** | **W m^-2^** |
| 1 | 9 | 2.54 | 2.3±0.13 | 19±1.04 | 9.2±0.51 |
| 1 | 9 | 3.67 | 3.63±0.09 | 29±0.72 | 9.9±0.25 |
| 1 | 9 | 5.09 | 5.1±0.21 | 41±1.68 | 10.1±0.41 |
| 1 | 9 | 5.09 | 5.1±0.22 | 41±1.68 | 5.7±0.23 |
| 2 | 18 | 5.09 | 5.6±0.11 | 44±0.88 | 3. ±0.06 |
| 3 | 27 | 5.09 | 6.0±0.16 | 48±1.28 | 2.23±0.06 |
